# Updates on Liquid Biopsy and ctDNA in Transplant Oncology

**DOI:** 10.3390/cancers17121930

**Published:** 2025-06-10

**Authors:** Abigail Loszko, Matthew M. Byrne, Cristina Jimenez-Soto, Koji Tomiyama, Yuki Bekki, Roberto Hernandez-Alejandro

**Affiliations:** 1 Department of Surgery, University of Rochester Medical Center, Rochester, NY 14642, USA; abigail_loszko@urmc.rochester.edu (A.L.); matthew_byrne@urmc.rochester.edu (M.M.B.); 2 Transplant Institute, University of Rochester Medical Center, Rochester, NY 14642, USA; cristina_jimenezsoto@urmc.rochester.edu (C.J.-S.); koji_tomiyama@urmc.rochester.edu (K.T.); yuki_bekki@urmc.rochester.edu (Y.B.)

**Keywords:** liquid biopsy, transplant oncology, ctDNA, circulating tumor DNA, next-generation sequencing, liver transplantation, hepatobiliary malignancy, hepatocellular carcinoma, cholangiocarcinoma, colorectal liver metastasis

## Abstract

Transplant oncology is a rapidly evolving discipline that incorporates oncology, transplant medicine, and surgery. As the field continues to grow, there remains an opportunity to enhance patient selection, detect recurrence after liver transplantation, and optimize treatment after recurrence. Liquid biopsies are an emerging resource to improve patient care. In this review, we evaluate the most recent available data on circulating tumor DNA and how it pertains to primary and metastatic hepatobiliary malignancies. We discuss the opportunities and current limitations to clinical practice, especially in relation to total hepatectomy and liver transplantation. We conclude that as both transplant oncology and our understanding of circulating tumor DNA continue to evolve, rigorous, prospective study is required to integrate this technology into a clinical paradigm.

## Simple Summary

As the field of transplant oncology continues to grow, so does the necessity for minimally invasive and reliable biomarkers for patient selection, recurrence detection, and analysis of treatment effects. Circulating tumor DNA is an emerging biomarker with growing data from the surgical oncology field. We review the current status of ctDNA as it pertains to transplant oncology for primary and metastatic hepatobiliary malignancies and acknowledge the need for transplant oncology centers to investigate and report on their experience with this novel biomarker.

## 1. Introduction

### 1.1. Transplant Oncology

Transplant oncology is a rapidly evolving discipline that incorporates oncology, transplant medicine, and surgery. Liver transplantation (LT) for malignant indications has gained traction over the past three decades, providing the only potentially curative treatment for patients with unresectable primary or secondary hepatobiliary malignancies, such as hepatocellular carcinoma (HCC), cholangiocarcinoma (CCA), and colorectal liver metastases (CRLM) [1,2].

As the field continues to grow, there remains an opportunity to enhance patient selection, detect recurrence after LT, and optimize treatment after recurrence [3]. Poor outcomes related to high rates of recurrence among these patients can be mitigated with the early detection and targeted treatment of recurrence post-transplant, but current diagnostic methods for detecting this recurrence are lacking. Liquid biopsies are an emerging resource with much promise to improve patient selection and post-transplant monitoring. In this review, we summarize recent evidence on ctDNA assays, their applications in transplant oncology for hepatic malignancies, and how they inform patient care in HCC, CCA, and CRLM.

### 1.2. Liquid Biopsy and ctDNA

Liquid biopsies consist of tumor-derived analytes that can be identified in a patient’s body fluids, such as blood, saliva, or urine. These analytes can be shed by both primary and metastatic tumors and include circulating tumor DNA (ctDNA), circulating tumor cells (CTCs), cell-free DNA (cfDNA), circulating cell-free RNA (ccfRNA), and extracellular vesicles (exosomes) containing subcellular components such as nucleic acids and proteins [4,5,6,7]. Among these, plasma-derived ctDNA has emerged as the most utilized blood-based biomarker [4] and will be the primary focus of this review, given its growing role in transplant oncology.

ctDNA refers to fragments of tumor-derived DNA that enter the systemic circulation primarily through passive mechanisms such as lysis, apoptosis, and necrosis, but active secretion is also believed to play a role [4,8,9]. ctDNA generally comprises less than <0.1% to 10% of total cfDNA, with the remainder originating from the turnover of normal host cells [4,10]. This low and variable concentration, combined with high fragmentation and relatively short size, poses significant challenges for ctDNA detection and quantification [4,9,10].

The two main approaches used for ctDNA detection are polymerase chain reaction (PCR)-based and next-generation sequencing (NGS)-based methods. PCR techniques, such as digital droplet PCR (ddPCR) and BEAMing (Beads, emulsion, amplification, and magnetics), are sensitive but also limited to analyzing known mutations in small quantities. In contrast, NGS allows for the comprehensive profiling of known and novel mutations [11]. Beyond detecting specific mutations and genomic alterations, ctDNA analysis can also quantify ctDNA levels, offering a promising avenue for cancer diagnosis and monitoring [11]. Among the literature and herein, the presence or absence of tumor mutations on ctDNA assays is typically reported as a “positive” or “negative” result. Quantitative ctDNA data, on the other hand, are often expressed as the variant allele fraction (VAF) or as the absolute number of mutant molecules per unit volume of plasma. Additionally, the overall burden of mutations in ctDNA can be assessed and reported as the tumor mutational burden (TMB).

#### 1.2.1. Tumor Informed vs. Agnostic

ctDNA approaches can be further differentiated by whether they are tumor-informed or tumor-agnostic. For tumor-informed assays, prevalent mutations are identified from a patient’s tumor tissue to develop a personalized gene panel for subsequent ctDNA monitoring in the blood [12]. They generally offer higher sensitivity and specificity given their targeting of known tumor-specific mutations for each patient [13]. Tumor-informed assays are particularly ideal for minimal residual disease detection (MRD) and recurrence monitoring but require each patient to have first undergone a biopsy or surgical resection of the tumor of interest [14]. Such assays, however, are also limited in their ability to detect new mutations or new secondary primary tumors that may develop after the initial tumor sample was obtained.

In contrast, tumor-agnostic assays (also referred to as tumor-uninformed or tumor-naïve assays) do not rely on prior knowledge of a patient’s specific tumor mutations and instead utilize the same predefined panel of common cancer-associated genomic or epigenomic alterations for every patient [12]. Tumor-agnostic assays are thus logistically advantageous when access to tumor tissue is not feasible and are further associated with faster turnaround time and decreased cost [13]. Nonetheless, the convenience of a broad, pre-selected panel is traded for decreased sensitivity, given that patient-specific alterations may not be captured with enough coverage [14].

In transplant oncology, we envision tumor-informed ctDNA analyses to be the more practical tool. Logistically, utilizing a diagnostic biopsy or previous surgical resection will be the available tissue in the pre-transplant setting—without having patients undergo a biopsy for ctDNA purposes only. In the post-transplant setting, the total hepatectomy specimen provides adequate tissue for tumor-informed mapping. This may be especially useful for metastatic lesions with multiple cites of foci with variance in tumor intrinsic biology [15].

#### 1.2.2. Current Status in Clinical Use

Multiple commercially available assays have been developed for detecting ctDNA of solid tumors. These assays continued to be widely represented in ongoing clinical trials, with a few having received full Food and Drug Administration (FDA) approval or special designations for clinical use in certain indications. In 2020, the commercial assays Guardant360^®^ CDx and FoundationOne^®^ Liquid CDx received FDA approval for comprehensive genomic profiling of advanced solid tumors to inform targeted treatment of actionable mutations [16,17].

More recently, in July 2024, Guardant Health’s Shield™ became the first ctDNA assay to receive FDA approval as a primary colorectal cancer screening tool among average-risk individuals [18,19]. This approval was based on a prospective trial demonstrating high sensitivity and specificity for cancer detection, although its performance in detecting precancerous lesions was much more limited [20]. While no commercial liquid biopsy assay has yet received full FDA approval for MRD detection of solid tumors, Signatera™ and FoundationOne^®^ Tracker have both received FDA Breakthrough Device Designation for this indication [21,22].

Notably, many commercially available tumor-agnostic assays have already broadened their gene panels and are expected to continue doing so in response to the growing evidence supporting targeted therapies and rising demand for comprehensive tumor profiling and MRD detection. For example, Guardant360^®^ CDx and FoundationOne^®^ Liquid CDx have expanded from 55 and 311 genes at FDA approval to 74 and 324 genes, respectively [23], while even broader assays like Guardant360^®^ Liquid and LabCorp^®^ Plasma Complete have grown to 739 [24] and 521 [25] genes.

## 2. Advantages of Liquid Biopsy

### 2.1. Minimally Invasive

The obvious advantage that liquid biopsies have over conventional tissue biopsies is that they are inherently less invasive. Not only do liquid biopsies increase convenience but they also decrease overall risks of additional tumor biopsies, such as excessive internal bleeding, damage to surrounding anatomical structures, or disease dissemination. While tumor-informed ctDNA assays still require an initial tumor tissue sample, subsequent dynamic and detailed information about tumor biology can be provided solely from a blood draw, without the need for follow-up tissue biopsies. Furthermore, tumor-agnostic assays provide an option to eliminate the need for biopsy altogether, which can be especially beneficial in population screening, cases with hard-to-reach tumors (where the risk of biopsy is not worth the benefit), or when the initial attempt at tissue biopsy was non-diagnostic/not sufficient.

### 2.2. Improved Capture of Tumor Heterogeneity

One of the most compelling advantages of ctDNA is its ability to reflect the complex and evolving molecular landscape of hepatobiliary malignancies. Traditional tissue biopsies provide only a snapshot of the tumor, often limited to a single site, and may fail to capture intratumoral or spatial heterogeneity [26]. In contrast, ctDNA integrates genetic material shed from multiple regions of the primary tumor and metastatic sites, potentially offering a more comprehensive view of tumor heterogeneity [27].

For hepatocellular carcinoma (HCC) and cholangiocarcinoma (CCA), where the genomic landscape can be highly diverse and unstable, ctDNA can uncover mutations that might be missed by single-site sampling. Studies have demonstrated discordance between tissue biopsies and plasma ctDNA, suggesting that ctDNA can detect mutations arising from different tumor subclones [28,29]. This is particularly relevant as tumors evolve under selective pressures such as locoregional therapies, systemic treatments, or immunosuppression post-transplant [5].

Moreover, ctDNA analysis allows longitudinal monitoring, enabling clinicians to track emerging mutations, clonal evolution, and resistance mechanisms over time [5]. This dynamic surveillance supports personalized treatment strategies—such as adapting systemic therapies based on newly acquired mutations (e.g., IDH1/2, FGFR2 in CCA [30,31], or TP53 alterations in HCC) or identifying actionable targets for adjuvant therapy in the transplant setting.

By better reflecting the dynamic and diverse genomic landscape of hepatobiliary malignancies, ctDNA facilitates more personalized treatment approaches. This enables tailored therapy selection based on a patient’s evolving mutational profile and allows clinicians to adjust treatment strategies in response to newly identified mutations, ultimately improving disease management and outcomes.

### 2.3. Increased Sensitivity and Specificity Compared to Traditional Surveillance Methods

While the currently accepted methods for surveillance include interval biomarker screening and cross-sectional imaging with computed tomography (CT), magnetic resonance imaging (MRI), or positron emission tomography (PET) [32,33,34,35], this approach is far from perfect. Imaging modalities often fall short in detecting MRD or early recurrence, as they offer only static views and lack real-time insight into tumor dynamics. Evidence has also shown that glycoprotein-based tumor markers like alpha-fetoprotein (AFP), carbohydrate antigen 19-9 (CA-19-9), or carcinoembryonic antigen (CEA) lack sensitivity and specificity [36,37]. AFP especially has come under scrutiny as a reliable biomarker given only 50–70% of HCC tumors secrete AFP, thus limiting its utility in early recurrence [29,38].

In theory, ctDNA can be intrinsically more specific than commonly used serum biomarkers because its detection is based on genetic changes present in the cancer cells [29]. Additionally, ctDNA’s ability to provide a real-time assessment of tumor biology with increased specificity is strengthened by having a short half-life in the circulation system (range of minutes to hours) [39,40,41,42]. Serum protein biomarkers, however, have relatively long half-lives (days to weeks), which may correspond with persistently elevated concentrations even after tumor removal [42,43].

## 3. Proposed Applications of ctDNA in Transplant Oncology (Figure 1)

### 3.1. Earlier Detection and Diagnosis

ctDNA may enable earlier diagnosis of malignancies, often before radiographic or symptomatic manifestations. In transplant oncology, this could translate to identifying cancers at more treatable stages—potentially before metastatic spread—thereby improving outcomes and alleviating the need for transplantation. Unlike conventional imaging or serum tumor markers (e.g., AFP, CEA), ctDNA assays offer greater sensitivity and specificity, which may eventually render some traditional diagnostics obsolete. Moreover, in high-risk populations (e.g., those with chronic liver disease or cirrhosis), ctDNA could serve as a screening tool [38,44]. Nevertheless, its use must be balanced against the risks of overdiagnosis and false positives, which can lead to unnecessary interventions or patient anxiety [45].

**Figure 1:**
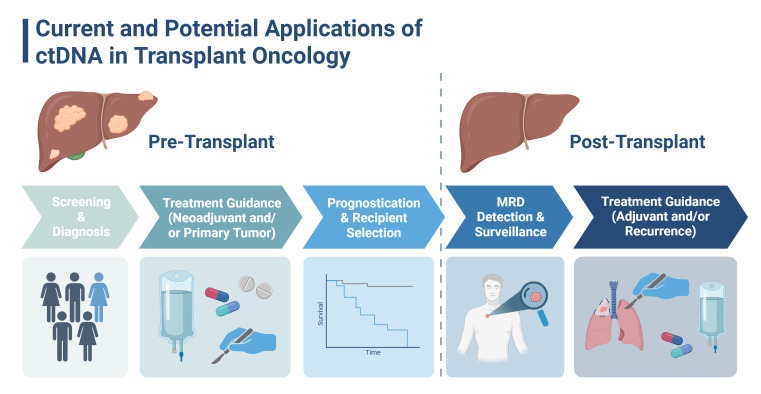
Current and potential applications of circulating tumor DNA (ctDNA) in transplant oncology for primary and secondary hepatic malignancies. Minimal residual disease (MRD). Figure created with Biorender.com.

### 3.2. Risk Stratification and Patient Selection

The effective treatment of hepatobiliary malignancy requires a multidisciplinary and multimodality approach to appropriate patient selection [2]. Pre-transplant ctDNA profiling offers an additional tool to risk stratify and select patients for liver transplantation. This molecular-level insight can refine transplant eligibility by identifying those with more aggressive phenotypes or higher metastatic potential [46], who may otherwise appear suitable by conventional criteria but are at elevated risk of recurrence.

This may be possible based on a careful analysis of prognostic factors. ctDNA levels have shown a correlation with tumor burden, stage, and vascular invasion [42,47,48,49,50,51,52,53,54,55,56,57]. There is not enough evidence to know whether higher pre-treatment or pre-transplant ctDNA levels are associated with worse outcomes. We hypothesize that ctDNA clearance (conversion from positive to negative ctDNA result) post-transplant may portend a better prognosis, given that ctDNA would only be expected to persist in systemic circulation if residual disease were present. Thus, ctDNA could serve as a dynamic prognostic biomarker that reflects both baseline disease aggressiveness and response to therapy.

### 3.3. Post-Transplant MRD Detection, Surveillance, and Recurrence Monitoring

Perhaps the most impactful application of ctDNA in transplant oncology lies in post-transplant surveillance. The detection of minimal residual disease (MRD) or early molecular recurrence—before it becomes radiographically evident—can facilitate prompt, targeted intervention and improve outcomes. This has been shown to provide evidence of recurrence at an earlier timepoint [58]. ctDNA surveillance may be especially useful in high-risk patients and could complement or even surpass conventional imaging and biomarker-based monitoring in sensitivity and lead-time advantage.

In transplant oncology, the role of post-transplantation systemic therapy is yet to be understood. The landmark TransMet trial showed in CRLM that liver transplantation plus chemotherapy had improved survival over chemotherapy alone [59]. Other centers in Europe and the United States have not routinely utilized chemotherapy post-transplant in patients with CRLM [60,61]. In HCC and CCA, other adjunctive therapies, including immunotherapy, have been proposed as an adjunct systemic treatment in the peri-transplant phase [62,63]. Early reports have shown a high risk of acute graft rejection when treated with immune checkpoint inhibitors [64], but this was not seen in patients treated with tyrosine kinase inhibitors [65]. ctDNA may provide insight into which patients may benefit from post-transplant systemic therapy, but the data to determine this are not currently available.

### 3.4. Obstacles to Clinical Implementation

Despite its potential, ctDNA-based testing in transplant oncology faces several limitations. Technical issues include low ctDNA quantity, fragment heterogeneity, and limited stability, which complicate detection and interpretation—especially in early-stage cancers or after neoadjuvant therapy [66]. Subclonal mutations with low allele frequencies are often missed, and intratumoral heterogeneity further reduces the reliability of a single ctDNA sample [67,68]. These challenges are compounded by the lack of standardized, sensitive, and specific assays, and the predominance of retrospective, proof-of-concept studies that have yet to be widely validated [38,69].

Pre-analytical variables—such as blood collection, handling, and storage—introduce substantial variability and are a major source of error, accounting for up to 68% of testing inaccuracies [5,70]. Differences in sample processing, assay platforms, and reporting protocols limit clinical integration and cross-study comparison [11,38]. Additionally, false negatives are common in low-shedding tumors or cases with minimal residual disease, where current assays may lack the sensitivity to detect small or short ctDNA fragments [71].

Finally, practical limitations include high costs, inconsistent global availability, and the absence of consensus guidelines on testing intervals or clinical interpretation. Non-tumor cfDNA (potentially from the donor of the transplanted organ) and biological noise from cell death processes (which is inherently increased in the immediate postoperative period) also complicate result analysis [72,73]. Without broader standardization across analytical workflows and regulatory frameworks, the routine use of ctDNA in transplant hepatology remains constrained.

## 4. Current Evidence

### 4.1. Hepatocellular Carcinoma (HCC)

Liver cancer is the third leading cause of cancer-related deaths worldwide, with hepatocellular carcinoma (HCC) comprising the majority of cases (75–85%) [74,75]. It has become one of the leading indications for transplant, representing the primary diagnosis for 10.3% of waitlisted candidates and 10.4% of transplanted recipients in the United States in 2023 [76]. Despite strict adherence to morphologic criteria in selecting transplant recipients, the recurrence rate of HCC after transplant is still around 6–18% [77]. Maximizing outcomes among these patients is contingent on early detection, careful selection, and postoperative surveillance, and multiple studies have highlighted the utility of ctDNA for the perioperative management of HCC patients in these areas (Table 1).

#### 4.1.1. Diagnosis and Screening

HCC often arises in patients with cirrhosis or chronic hepatitis B, making this population a key target for screening. Although early detection significantly improves survival, fewer than 20% of at-risk individuals undergo regular surveillance, leading to late-stage diagnoses and consequently worse outcomes [11]. The current guidelines recommend imaging-based screening every six months [78,79,80,81], but limitations in sensitivity and accessibility persist. ctDNA and cfDNA—particularly TERT promoter mutations—are emerging as promising noninvasive biomarkers that could enhance early detection in high-risk patients and complement existing surveillance strategies [38,44].

**Table 1:** Current evidence of ctDNA testing among patients undergoing surgical treatment of hepatocellular carcinoma (2022–2025).

Study (Country)	Cancer (n)	Surgical Procedure	Assay Type/ Biomarkers	NSG/ ddPCR	Tumor- Informed/ -Agnostic	Time Point(s)	Relevant Findings
Abdelrahim et al., 2024 * [82] (USA)	HCC (111)	Resection **Transplant**	Signatera^TM^ (16 bespoke variants)	NGS	Informed	Post-surgery	Among 36 LT patients with available MRD testing, all were ctDNA(−) In resection cohort, ctDNA(+) prognostic for poor RFS (HR: 12, *p* < 0.0001)
Wehrle et al., 2024 [83] (USA)	HCC (47)	Resection **Transplant**	Guardant 360^®^ (83-gene panel)	NGS	Agnostic	Post-surgery	Identifiable TMB on postoperative ctDNA predicts HCC recurrence and outperformed AFP
Jiang et al., 2022 * [49] (China)	HCC (45)	**Transplant**	328-gene panel	--	--	Pre-surgery	ctDNA(+) before LT strongly associated with a remarkably augmented RR (48.6% vs 0%) and decreased DFS
Zhao et al., 2022 [84] (China)	HCC (66)	Resection **Transplant**	Universal panel, 15 bespoke variants	NGS	Agnostic and Informed	Post-surgery	ctDNA(+) after resection/LT strongly associated with worse RFS CTC status was complementary to ctDNA status, and the combination improved MRD detection and recurrence prediction
Xu et al., 2023 [85] (China)	HCC (20)	Resection	13-gene panel	NGS	Agnostic	Post-surgery	MRD positivity had sensitivity of 75% and specificity of 100% for early recurrence RD positivity was independent predictor of poor RFS (HR: 13.00, *p* = 0.002)
Fu et al., 2022 [86] (China)	HCC (258)	Resection	150-gene panel + high-risk genes	NGS	Agnostic	Pre-surgery	Total mutant genes in ctDNA associated with early relapse (HR: 2.2, *p* < 0.001) High-risk patients had worse RFS (HR: 13.0, *p* < 0.001)

* abstract only; ctDNA—circulating tumor DNA; NGS—next-generation sequencing; ddPCR—digital droplet polymerase chain reaction; HCC—hepatocellular carcinoma; LT—liver transplant; MRD—minimal residual disease; RFS—recurrence-free survival; HR—hazard ratio; TMB—tumor mutational burden; AFP—alpha-fetoprotein; RR—recurrence rate; CTC—circulating tumor cell; DFS—disease-free survival.

#### 4.1.2. Correlation to Clinicopathologic Variables

In patients who underwent hepatectomy or liver transplantation for HCC, both pre- and postoperative ctDNA status consistently correlated with tumor differentiation, stage, the presence of microvascular invasion, and portal venous thrombus [42,47,48,49,50,51,52]. The correlation was not as consistent for tumor size or number. While preoperative ctDNA status did correlate significantly with larger tumor size and multiple tumor lesions in a study of patients undergoing hepatectomy by Wang et al. [47], there was no such correlation in studies by Fu et al. [86] or Liao et al. [50].

#### 4.1.3. Prognosis and Surveillance

In transplant oncology, the opportunity to detect HCC recurrence after transplantation may be the most important use. From the resection data, patients with detectable ctDNA postoperatively are at a significantly higher risk of recurrence or metastasis [42,47,50,84,85,87]. While data are limited, there is initial evidence to support the prognostic value of ctDNA after transplantation [48,82,88].

Data on the clearance of ctDNA after transplant are limited, given the lack of samples analyzed for ctDNA prior to transplant in the reported studies. In the study by Hong et al., only two transplanted HCC patients had both pre- and post-transplant ctDNA results. Both were ctDNA-positive pre-transplant. One cleared ctDNA post-transplant and remained recurrence-free during follow-up, but so did the patient who remained ctDNA-positive, making it difficult to interpret the significance of ctDNA clearance [88]. Moreover, while no pre-transplant ctDNA analysis was reported for the patients in the study by Abdelrahim et al., all thirty-six patients who had ctDNA results in the post-transplant MRD window were ctDNA-negative [82], suggesting the eradication of disease with the removal of the liver. One patient later turned positive on serial testing, with subsequent disease-related death 1 year from ctDNA positivity. Overall, ctDNA may provide some benefit for prognostication in patients transplanted for HCC, but further work is required to definitively utilize ctDNA as a post-transplant prognostic tool. 

#### 4.1.4. Comparison to Traditional Surveillance

After curative surgical treatment, surveillance imaging with contrast-enhanced CT or MRI at regular intervals is a commonly accepted practice [89]. Zhao et al. showed that a positive circulating tumor cell (CTC) and ctDNA improved the median lead time of recurrence detection by more than 3 months over CT or MRI [84], but more work will be required to understand the role of lead-time bias in the utilization of these biomarkers [90].

Often used in conjunction with imaging, alpha-fetoprotein (AFP) remains the most widely used biomarker for hepatocellular carcinoma (HCC), but its diagnostic, prognostic, and surveillance capabilities are limited. Secreted by only 50–70% of HCC tumors [29,38,91], AFP fails to identify a significant proportion of patients with active disease. Its modest sensitivity (50–60%) and specificity are further compromised by fluctuations due to cirrhosis, hepatitis, or antiviral therapy [83,92,93]. AFP performs poorly in early-stage HCC and struggles to detect small lesions or predict recurrence following curative interventions like resection or transplant [93]. While high AFP levels are linked with advanced disease and vascular invasion, its inability to detect recurrence early or capture tumor heterogeneity renders it suboptimal as a stand-alone tool [92].

ctDNA, on the other hand, has shown superior performance over AFP and des-gamma-carboxy prothrombin (DCP) in detecting MRD and predicting recurrence after resection or transplantation [42,47,83,84,94]. However, findings such as those by Huang et al. suggest that AFP and DCP may still offer better predictive value for post-transplant recurrence, indicating that ctDNA alone may not yet replace traditional methods. Emerging evidence supports combining ctDNA with protein biomarkers to enhance sensitivity and improve early detection [42].

### 4.2. Cholangiocarcinoma (CCA)

Cholangiocarcinoma is less common than HCC, with intrahepatic cholangiocarcinoma (iCCA) accounting for 15% of primary hepatic malignancies [74,75]. Overall, CCA has an insidious presentation with high morbidity and mortality related to late-stage diagnosis. LT for CCA is focused on hilar (hCCA) and intrahepatic (iCCA) disease and is currently being conducted with caution for highly selected patients [95,96]. LT for CCA is an emerging field and there are limited data available on the utility of where ctDNA may play a role in this indication (Table 2).

#### 4.2.1. Correlation to Clinicopathologic Variables

The data available for ctDNA utilization in CCA are primarily derived from the resection data. In 2021, Wintachai et al. reported that variant allele frequency (VAF) in ctDNA of CCA patients correlated with tumor staging [97]. In a more recent study by Yoo et al. that reported on a sub-analysis of the phase II STAMP trial, ctDNA positivity was observed to increase with higher stage in CCA patients. Specifically, ctDNA positivity rates were reported as 16.2% for stage II, 27.5% for stage III, and 41.6% for stage IV [98]. In the stage IV group, ctDNA positivity also correlated significantly with higher lymph node status and R1 resection margins [98].

#### 4.2.2. Prognosis and Surveillance

A sub-analysis of the phase II STAMP trial has also shown that positive ctDNA status is predictive of worse disease-free survival (DFS) after resection. ctDNA positivity during the molecular residual disease (MRD) window (post-surgery and before adjuvant chemotherapy) was significantly associated with inferior DFS (HR 1.8; 95% CI 1.06–3.07; *p* = 0.029) compared to ctDNA-negative patients. After the MRD window, ctDNA presence at any time post-surgery was associated with significantly worse DFS (HR 3.81; 95% CI 2.22–6.54; *p* <0.001) and a remarkably high recurrence rate of 95.7% [98].

While there is not enough evidence to apply the prognostic findings of the STAMP trial to transplant oncology, the study by Hong et al. offers limited but supportive evidence for the utility of ctDNA in post-transplant surveillance of CCA. Among three patients who underwent transplant for CCA with available post-transplant ctDNA data, two were ctDNA-negative post-transplant (including one who converted from pre-transplant positivity) and both remained recurrence-free during follow-up. In contrast, one patient with post-transplant ctDNA positivity developed recurrence and died within the follow-up period [88].

#### 4.2.3. Comparison to Traditional Surveillance

Among the eleven patients in the STAMP trial with ctDNA that became newly positive during adjuvant treatment, all experienced radiological recurrence [98]. Notably, ctDNA positivity occurred in the setting of normal CA 19-9 levels for five of these patients, with a mean lead time of 5.8 months prior to radiological recurrence. ctDNA positivity in the presence of normal CEA occurred in three patients, with a mean lead time of 7.4 months before evidence of radiological recurrence [98]. While these findings highlight ctDNA’s ability to detect recurrent disease during surveillance prior to imaging and traditional biomarkers, no algorithm has been proposed for how to address ctDNA positivity despite otherwise negative imaging and serum tumor markers. There are few published reports in the literature that describe a successful change in adjuvant therapy guided by ctDNA positivity [99,100], but further prospective trials will need to be pursued to determine if ctDNA-guided treatment decisions would have an impact on patient survival.

Given the aggressive nature of CCA and the caution utilized in LT for CCA, ctDNA positivity prior to LT may be a useful adjunct in the risk assessment of potential transplant candidates. After LT, ctDNA positivity may warrant further workup or initiation of adjuvant systemic therapy, but the data for these recommendations are currently not available.

**Table 2:** Current evidence of ctDNA testing among patients undergoing surgical treatment of cholangiocarcinoma and other hepatobiliary malignancies (2022–2025).

Study (Country)	Cancer (n)	Surgical Procedure	Assay Type/ Biomarkers	NSG/ ddPCR	Tumor- Informed/ -Agnostic	Time Point(s)	Relevant Findings
Hong et al., 2024 [88] (USA)	HCC (9) CRLM (8) CCA (3) cHCC/CCA (1)	**Transplant**	Guardant 360^®^ (83-gene panel) [All] Guardant 360 CDx^®^ [HCC only] Guardant Reveal^TM^ [CRLM only]	NGS	Agnostic	Pre-nACT, Pre- and Post-surgery	ctDNA clearance (+/−) occurred in 50% of patients after LT Higher absolute RR in patients who had ctDNA(+) after LT (50%) compared to those who did not (25%) (*p* = 0.367)
Huang et al., 2024 [48] (China)	HCC (67) iCCA (7)	**Transplant**	Somatic mutations (SNV & CNV)	NGS	Informed	Pre- and Post- surgery	ctDNA(+) status pre- and post-LT associated with higher RR, shorter RFS ctDNA increased upon recurrence while AFP, DCP remained negative in 2 patients
Yoo et al., 2025 [98] (Korea)	eCCA (89)	Resection	Signatera^TM^ (16 bespoke variants)	NGS	Informed	Post-surgery (Pre-, During, and Post-ACT)	45.5% (5/11) had ctDNA turn (+) before imaging (mean lead time: 174d) while CA19-9 remained normal 27.3% (3/11) had ctDNA turn (+) before imaging (mean lead time: 222d), while CEA remained normal ctDNA(+) any time post-surgery had worse DFS (HR: 3.81, *p* < 0.001), higher RR (95.7% vs. 47.6%), inferior OS
Kim et al., 2023 [101] (Korea)	eCCA (14) iCCA (4)	Resection	118-gene panel	NGS	Informed	Pre- and Post- surgery	Postoperative plasma mutations detected recurrence or metastasis with 44% sensitivity and 45% specificity Postoperative ctDNA from 50% (8/16) was (+) for new somatic mutations not present in resection specimen

ctDNA—circulating tumor DNA; NGS—next-generation sequencing; ddPCR—digital droplet polymerase chain reaction; HCC—hepatocellular carcinoma; CRLM—colorectal liver metastasis; CCA—cholangiocarcinoma; cHCC/CCA—combined hepatocellular carcinoma/cholangiocarcinoma; nACT—neoadjuvant chemotherapy; LT—liver transplant; RRrecurrence rate; iCCA—intrahepatic cholangiocarcinoma; SNV—single nucleotide variant; CNV—copy number variant; RFS—recurrence-free survival; AFP—alpha-fetoprotein; DCP—des-gamma-carboxy prothrombin; eCCA—extrahepatic cholangiocarcinoma; ACT—adjuvant chemotherapy; CA 19-9—carbohydrate antigen 19-9; CEA—carcinoembryonic antigen; DFS—disease-free survival; HR—hazard ratio; OS—overall survival.

### 4.3. Colorectal Liver Metastases (CRLM)

Colorectal cancer (CRC) is the third most common cancer worldwide, and around 50% of these patients will develop CRLM. Of these patients, 20% present with metastasis at the time of presentation and 80% of patients with metastatic disease are unresectable [102]. As the incidence of CRC rises in younger populations [103], so will the necessity for liver transplantation in patients with liver-limited metastatic disease. ctDNA has the potential to impact the perioperative management of this population (Table 3).

#### 4.3.1. Correlation to Clinicopathologic Variables

ctDNA is well-correlated with many clinicopathologic variables that are already established prognostic factors in patients with CRLM. Both pre- and postoperative ctDNA is consistently reported in the literature as correlating positively with markers of high tumor burden, such as tumor size, number of metastases, or Clinical Risk Score (CRS) [53,54,55,56,57]. It has also been shown to be associated with other tumor characteristics such as decreased differentiation [104], synchronous and bilobar metastases, and extrahepatic recurrence [57,105]. Furthermore, ctDNA status after resection shows a strong inverse correlation with a favorable pathologic response to chemotherapy [106,107].

#### 4.3.2. Prognosis and Surveillance

In patients undergoing resection for CRLM, preoperative ctDNA positivity has been linked to higher recurrence risk and poorer survival outcomes, including shorter DFS and overall survival (OS) [53,55,104,105,108], although not all studies have confirmed this association. Newhook et al. found no correlation between preoperative ctDNA and prognosis, which they state may be related to the confounding effects of neoadjuvant chemotherapy [109]. Other studies support this, suggesting that chemotherapy may lower ctDNA levels, weakening its predictive value if measured only once preoperatively [54,107,110]. Some evidence indicates that ctDNA dynamics, such as clearance after treatment, may be more predictive than a single preoperative result [54,56,107,109]. Given the limited data available in the pre-transplantation setting, ctDNA positivity (or negativity) prior to transplant should not limit patient selection by other tumor biology-driven methods [111,112,113]. In the post-resection literature, ctDNA positivity consistently correlates with worse prognosis, including significantly higher recurrence rates and shorter DFS/RFS. Studies report recurrence rates as high as 94% in post-op ctDNA-positive patients vs. 31–44% in ctDNA-negative individuals [56,57]. The positive predictive value (PPV) of post-op ctDNA for recurrence is often very high—up to 100% in some cohorts—and associated hazard ratios (HRs) for RFS range from 3.3 to 7.6 [54,56,57,106,108,109,110,114,115,116,117,118,119]. These findings hold even after adjusting for clinicopathological factors. However, recurrences have occurred despite negative ctDNA, likely due to assay sensitivity limitations or minimal tumor shedding, emphasizing the need for serial testing and complementary biomarkers [106,110,114,115,117].

In the transplant context, ctDNA is likely best used to enhance post-transplant surveillance. Persistent post-transplant clearance likely indicates effective tumor removal. Reports from the Cleaveland Clinic on a small cohort of patients who underwent transplant for CRLM have shown high rates of ctDNA clearance post-liver transplant, whereas persistently detectable ctDNA may warrant closer follow-up and possible adjuvant treatment [83,88,120]. A subgroup analysis of the CIRCULATE-Japan GALAXY trial showed survival benefit when ctDNA was used to guide adjuvant chemotherapy after resection of CRLM [58]. While transplanted patients are speculated to have a similar survival benefit from ctDNA-guided adjuvant chemotherapy, the exact relevance remains to be elucidated.

**Table 3:** Current evidence of ctDNA testing among patients undergoing surgical treatment of colorectal liver metastasis (2022–2025).

Author, Year (Country)	Cancer (n)	Surgical Procedure	Assay Type/ Biomarkers	NSG/ ddPCR	Tumor- Informed/ -Agnostic	Time Point(s)	Relevant Findings
Wehrle et al., 2023 [120] (USA)	CRLM (29)	Resection **Transplant**	Guardant360^®^ (83-gene panel)	NGS	Agnostic	Pre- and Post- surgery	Resection and LT associated with cleared ctDNA in patients who were ctDNA(+) before surgery (*p* = 0.009) Postoperative ctDNA associated with higher risk of recurrence (*p* = 0.042)
Kataoka et al., 2024 [58] (Japan)	CRLM (190)	Resection	Signatera™ (16 bespoke variants)	NGS	Informed	Pre- and Post- surgery	ctDNA positivity in the MRD window was 32.1% (61/190) ACT administered to 25.1% (48/190) In MRD-positive group, 24-month DFS was higher for patients treated with ACT (HR: 0.07, *p* < 0.0001)
Liu et al., 2024 [107] (China)	CRLM (114)	Resection	620-gene panel	NGS	Agnostic	Pre-nACT, Pre- and Post-surgery	ctDNA(+) at baseline and (−) after nACT had longer RFS (*p* = 0.001) and HRFS (*p* < 0.001) than those with ctDNA(+) persistently after nACT RFS (all *p* < 0.05) improved in patients ctDNA(−) after nACT (HR: 0.51, 95% CI 0.28–0.93), major pathologic response (HR: 0.34, 95% CI 0.19–0.62) and surgery combined with radiofrequency ablation (HR: 2.62, 95% CI 1.38–5.00)
Li et al., 2024 [57] (China)	CRLM (60)	Resection	Signatera™ (16 bespoke variants)	NGS	Informed	Pre- and Post- surgery, Post-ACT	Higher risk of recurrence in those with ctDNA(+) post-resection (HR: 4.8), post-ACT (HR, 6.0), (both, *p* < 0.001) Post-resection ctDNA(+) was only independent prognostic marker in multivariant analysis (HR: 5.1, *p* < 0.001)
Wang et al., 2023 [108] (China)	CRLM (34)	Resection	61-gene panel	NGS	Agnostic	Pre-nACT, Pre- and Post-surgery	Early changes in ctDNA but not CEA or CA19-9 were an independent indicator of RFS (HR: 4.0, *p* = 0.023)
Liu et al., 2023 [119] (China)	CRLM (134)	Resection	25-gene (J25) and 642-gene panels	NGS	Informed	Post-surgery	ctDNA(+) subgroup had shorter RFS (HR: 2.96, *p* < 0.05) ctDNA(+) patients who received ACT >2 months had longer RFS than those who received ≤2 months (HR: 0.377; 95% CI, 0.189–0.751; *p* < 0.05)
Newhook et al., 2023 [109] (USA)	CRLM (48)	Resection	Guardant^®^ variant classifier (23-gene panel)	NGS	Agnostic and Informed (for 38 patients)	Pre- and post-surgery	ctDNA(+) before and after surgery (+/+) associated with worse RFS (*p* = 0.001) ctDNA(+/−) associated with improved RFS and OS over ctDNA(+/+)
Marmorino et al., 2022 [118] (Italy)	CRLM (76)	Resection	24-gene panel	ddPCR	Informed	Post-surgery	ctDNA(+) patients had shorter RFS than ctDNA(−) (median RFS 12.7 vs. 27.4, HR: 2.09, *p* = 0.008).
Nishioka et al., 2022 [117] (USA)	CRLM (105)	Resection	70-gene panel	NGS	Agnostic	Post-surgery	ctDNA(+) within 180 days was the only independent risk factor on multivariate analysis for recurrence at 1 year (94% vs. 49%; HR: 11.8, *p* = 0.003)
Ogaard et al., 2022 [110] (Denmark)	CRLM (96)	Resection	Methylation profile/C9orf50, CLIP4, KCNQ5	ddPCR	Agnostic	Pre- and Post-surgery, Post-ACT	Patients with ctDNA(+) postoperatively or post-ACT had lower RFS than patients with ctDNA(−) (HR: 4.5, *p* < 0.0001, HR: 8.4, *p* < 0.0001) ctDNA(+) detected before radiological recurrence in 55.6% of ctDNA(+) patients, with median 3.1-month lead time. ctDNA status at the time of inconclusive imaging predicted recurrence with PPV and NPV of 100%, and 75%, respectively (*p* = 0.0003).

ctDNA—circulating tumor DNA; NGS—next-generation sequencing; ddPCR—digital droplet polymerase chain reaction; CRLM—colorectal liver metastasis; LT—liver transplant; MRD—minimal residual disease; ACT—adjuvant chemotherapy; DFS—disease-free survival; HR—hazard ratio; nACT—neoadjuvant chemotherapy; RFS—recurrence-free survival; HRFS—hepatic recurrence-free survival; CI—confidence interval; CEA—carcinoembryonic antigen; CA 19-9—carbohydrate antigen 19-9; OS—overall survival; PPV—positive predictive value; NPV—negative predictive value.

#### 4.3.3. Comparison to Traditional Surveillance

Common surveillance practices after curative surgical resection for CRLM consist of cross-sectional imaging and serum CEA levels. Compared to CEA, ctDNA has higher sensitivity in the detection of recurrence and is a stronger prognostic marker for RFS than CEA [108,114,115,116]. The significant advantage of ctDNA over standard-of-care imaging is the ability to detect molecular recurrence several months before clinical radiologic confirmation. Studies report median lead times of ctDNA detection over imaging ranging from 2.5 to 9 months [57,115,116]. While ctDNA still has yet to take the place of imaging in evaluating for recurrence after resection of CRLM, most studies mention its particular benefit as an adjunct to imaging that serves to support clinical decision making and reduce time to intervention [107,110,115]. As with HCC, more work will be required to understand the role of lead-time bias in the utilization of these biomarkers [90].

Finally, one limitation of ctDNA in the post-transplant setting is significantly decreased sensitivity in detecting recurrence in the lung with ctDNA positivity delayed until after confirmed radiographic evidence [57,110,115]. This is likely attributable to previously reported findings on how metastatic site influences tumor shedding in patients with metastatic colorectal cancer, with metastases of the lung and peritoneum associated with significantly lower ctDNA shedding than liver metastases [121].

## 5. Discussion

There has been a concurrent growth in the field of transplant oncology and liquid biopsy. Circulating tumor DNA (ctDNA) has generated considerable interest for its potential to enhance surveillance, diagnosis, recurrence detection, and treatment guidance in patients undergoing liver transplantation for hepatobiliary malignancies. The promise of a noninvasive, easily repeatable liquid biopsy that could provide real-time molecular insights and improve outcomes is particularly appealing. However, widespread clinical adoption remains limited, largely due to the need for further validation and standardization in transplant-specific settings.

Although the body of research surrounding ctDNA is steadily expanding, data specific to transplant recipients remain sparse. Some insights can be drawn from studies in patients undergoing hepatectomy, yet extrapolating these findings to the transplant setting must be performed cautiously. Key differences—such as the effects of post-transplant immunosuppression on tumor evolution and the potential interference of donor-derived cfDNA—complicate direct translation. Additionally, global implementation has been hindered by the absence of formal recommendations from transplant and oncologic societies and guideline committees. While some institutions have begun to adopt ctDNA protocols for peri-transplant monitoring [88], this remains the exception rather than the norm.

Transplant oncology has gained valuable insights from surgical oncology, particularly in refining selection criteria and post-transplant outcomes. However, not all approaches translate directly to transplant recipients. For example, adjuvant immunotherapy may offer benefits but also carries risks unique to this population, such as graft rejection. To determine whether ctDNA truly improves survival in patients transplanted for cancer, prospective multi-institutional studies are needed. As both ctDNA technologies and the field of transplant oncology continue to evolve, the integration of this promising tool will require further rigorous study and consensus development.

## 6. Conclusions

ctDNA holds immense promise across the transplant oncology continuum, from pre-transplant assessment through to post-transplant surveillance. As assay technologies improve and clinical utility becomes better validated in prospective studies, ctDNA may become a central pillar in the precision oncology approach to transplant candidates and recipients.

ctDNA in transplant oncology remains an emerging technology. There is an opportunity to further understand the underlying biological properties of tumor growth and secretion after transplantation and exposure to immunosuppression, as this is an area with limited basic and translational understanding. Furthermore, prospective studies and potential randomized controlled trials are urgently needed to understand the role of liquid biopsy in transplant oncology.

## Data Availability

No new data were created in the creation of this review.

## Abbreviations

The following abbreviations are used in this manuscript:

AFP	alpha-fetoprotein
BEAMing	Beads, emulsion, amplification, and magnetics
CA19-9	carbohydrate antigen 19-9
CCA	cholangiocarcinoma
ccfRNA	circulating cell-free ribonucleic acid
CEA	carcinoembryonic antigen
cfDNA	cell-free deoxyribonucleic acid
CRLM	colorectal liver metastases
CT	computed tomograph
CTC	circulating tumor cells
ctDNA	circulating tumor deoxyribonucleic acid
ddPCR	digital droplet polymerase chain reaction
DFS	disease-free survival
FDA	Food and Drug Administration
HCC	hepatocellular carcinoma
LT	liver transplantation
MRD	minimal residual disease detection
MRI	magnetic resonance imaging
NGS	next-generation sequencing
OS	overall survival
PCR	polymerase chain reaction
PET	positron emission tomograph
PFS	progression-free survival
RFS	recurrence-free survival
TMB	tumor mutational burden
VAF	variant allele fraction
